# Retraction: Long non-coding RNA PVT1 facilitates cell proliferation by epigenetically regulating FOXF1 in breast cancer

**DOI:** 10.1039/d2ra90085h

**Published:** 2022-09-05

**Authors:** Guangcheng Guo, Fang Wang, Mingli Han, Yuanting Gu, Xin Duan, Lin Li

**Affiliations:** Department of Breast Surgery, The First Affiliated Hospital of Zhengzhou University No. 1 Jian She East Road Zhengzhou 450052 China lilinlin126@sohu.com +86-0371-67967172

## Abstract

Retraction of ‘Long non-coding RNA PVT1 facilitates cell proliferation by epigenetically regulating FOXF1 in breast cancer’ by Guangcheng Guo *et al.*, *RSC Adv.*, 2018, **8**, 2740–2750, https://doi.org/10.1039/C7RA12042G.

The Royal Society of Chemistry hereby wholly retracts this *RSC Advances* article due to concerns with the reliability of the data. Images published in Fig. 2 and 6 of the article have been duplicated in another article.^1^ There are no common authors between the papers.

Rotated and scaled versions of the first and second tumor images in the ‘sh-con’ panel in Fig. 6B of this paper are duplicated in Fig. 5B of ref. 1.

A rotated version of the ‘si-con/MCF-7’ colony formation image in Fig. 2D of this paper is duplicated in Fig. 2E (‘si-con/SK-MEL-2’ image) of ref. 1. Some additional features can be observed in the image in Fig. 2D of this article, although the majority of the features overlap between the two images.

An inverted version of the ‘si-PVT1#2/MDA-MB-231’ colony formation image in Fig. 2D of this paper is duplicated in Fig. 2E (‘si-con/A375’ image) of ref. 1. Although some features do appear differently in each image, the majority of the features overlap between the two images.

The authors were asked to provide the raw data for this article, but did not respond. Given the significance of the concerns about the validity of the data, and the lack of raw data, the findings presented in this article are not reliable.

The authors were informed but have not responded to any correspondence regarding the retraction.

Signed: Laura Fisher, Executive Editor, *RSC Advances*

Date: 18th August 2022

## Supplementary Material
